# Continuous chains: childhood maltreatment and intimate partner violence victimization among displaced women in a war context

**DOI:** 10.1186/s12905-024-03156-2

**Published:** 2024-06-01

**Authors:** Hawkar Ibrahim, Katharina Goessmann, Frank Neuner, Benjamin Iffland

**Affiliations:** 1 Department of Clinical Psychology and Psychotherapy, Bielefeld University, Bielefeld, Germany; 2 vivo international e.V., Konstanz, Germany; 3 Department of Clinical Psychology and Violence Research, Bielefeld University, Bielefeld, Germany

**Keywords:** Intimate partner violence, Childhood maltreatment, Emotional violence, Childhood trauma, Armed conflict, Displaced women

## Abstract

**Background:**

Childhood victimization has been associated with long-term psychological effects and an increased risk of being victimized in later life. Previous research has primarily focused on sexual abuse during childhood, and a wide range of consequences have been identified. However, a significant gap remains in our understanding of the complex interaction between different forms of childhood abuse and violence in later life, particularly in the context of broader social stressors such as armed conflict and displacement.

**Methods:**

This study examines the association between exposure to different types of childhood maltreatment in the context of family and intimate partner violence (IPV) among displaced women living in refugee camps in northern Iraq. Structured interviews were conducted by trained female psychologists with 332 women aged between 20 and 62 years.

**Results:**

Results indicated that over one-third of the participating women reported experiencing at least one occurrence of IPV by their husbands within the past year. In addition, participants reported experiences of different types of maltreatment (physical, emotional, and sexual violence and physical and emotional neglect) perpetrated by family members in their childhood. While all forms of childhood maltreatment showed an association with IPV within the past year, only emotional childhood maltreatment was found to be a significant predictor of IPV in a multivariate analysis.

**Conclusion:**

The study highlights the ongoing impact of child maltreatment and its contribution to increased vulnerability to IPV victimization in later life. In addition, this study describes the specific cultural and contextual elements that contribute to IPV in refugee camps.

## Background

Violence against children is an escalating global problem with profound societal implications. It is estimated that nearly one billion children between the ages of 2 and 17 have experienced physical, sexual, or emotional violence or neglect in the past year [1]. Beyond the immediate impact of such violence, it is critical to understand the lasting effects on a child’s life trajectory.

A growing body of evidence suggests that early exposure to violence can have lasting effects, increasing the risk of victimization both in childhood and later in life [2, 3]. This phenomenon, referred to as revictimization, has been studied extensively, particularly with regard to the association between childhood sexual abuse and subsequent vulnerability in adulthood. Empirical evidence, such as Walker et al.‘s (2019) meta-analysis of 80 studies involving 12,252 survivors of child sexual abuse, suggests that nearly half of these individuals were sexually victimized later in life [4]. This is consistent with Classen et al. (2005), who concluded in a review of 90 studies that a history of childhood sexual abuse significantly increased the risk of later sexual victimization [5]. Yet the literature has focused disproportionally on sexual abuse in particular, often overshadowing other forms of child victimization, such as emotional abuse and neglect, particularly within the family [6–8]. In light of the profound impact of different forms of abuse on the holistic development of children, shaping their emotional, cognitive, and social trajectories in distinctive ways, analysing them separately might yield relevant insights into the complex interplay between violence, revictimization and mental health.

Accordingly, a growing body of studies presents evidence that various forms of childhood maltreatment are related to victimization later in life [9, 10]. In one of these studies, experiences of emotional abuse increased the odds of a later victimization in females by 10%, even when controlling for physical and sexual abuse [11]. Furthermore, the statistical association between physical maltreatment and later victimization diminished when controlling for experiences of emotional maltreatment. Accordingly, it has been suggested that the mechanisms linking early life adversities to later life victimization differ between sexual, physical, and emotional maltreatment. For emotional forms, it has been suggested that deficits in emotion processing impair individuals’ abilities to respond appropriately to social interactions and form healthy relationships [11, 12].

Current research on childhood maltreatment is biased towards high-income countries, which significantly marginalizes the experiences and challenges faced by people residing in low- and middle-income countries (LMICs). This is a gap in the scientific discourse, especially given that an estimated 85% of the world’s population lives in LMICs [13]. It is therefore not only essential, but urgent to attempt for a more comprehensive understanding of the long-term psychosocial effects of child maltreatment in contexts of different cultural traditions, societal norms, and histories in LMICs.

A critical dimension that is often overlooked is the long history of collective and structural forms of violence, i.e., war, civil conflict, and political instability that many LMICs have endured. These historical traumas have been shown to be strongly related to social attitudes, family relationships, and mental health [14–16]. It seems the aftermath of war and conflict goes beyond the immediate physical and infrastructural damage and can leave lasting psychosocial marks on survivors for decades [17].

The interplay between violence on the societal level and individual experiences of child maltreatment can potentially magnify the harmful effects on victims. However, most of the existing studies conducted in LMICs, especially those dealing with post-conflict situations, tend to focus either on the effects of displacement - which include changes in social roles, domestic and intimate partner violence (IPV), the stresses of daily life, the intricacies of residency status - or instead investigate the psychological consequences of war, focusing on mental health conditions such as depression, anxiety, or post-traumatic stress disorder (PTSD) [18–22]. Such a binary perspective, which primarily addresses either post-displacement conditions or trauma experienced during war and displacement, may unintentionally overlook the intricate relationships between non-war-related life experiences, such as childhood events, and the emergence of other psychosocial issues among war-affected populations. A more integrative and holistic approach is needed to understand the complexities that arise from the linking of childhood experiences with larger societal events and historical trauma. For example, little research has attempted to understand how cultural norms, family characteristics and structures in LMICs, particularly in war-affected regions, regarding age of marriage, polygamy, and kinship marriage contribute to the later victimization such as IPV of women who have been maltreated in childhood.

IPV is one of the most common forms of violence against women, occurring across various societal contexts, both in stable and conflict-affected regions [23, 24]. The World Health Organization (WHO) estimates that approximately one-third of women globally experience IPV at some point in their lives, either from current or former partners [25]. However, the ensuing disruption and social breakdown that accompanies armed conflict often exacerbate structural violence like pre-existing gender inequalities, which may put women at increased risk of interpersonal violence from their intimate partners [26]. In both conflict and post-conflict settings, degraded social infrastructure, attitudes about women’s roles, diminished security, and rising economic pressures seem to exacerbate women’s vulnerability to IPV [27–30].

In this context, when examining the impact of interpersonal trauma on women, it is important to consider polyvictimization, which refers to the experience of multiple forms of violence in different contexts and at different times, including childhood maltreatment, IPV, and other war and non-war related traumatic events [31]. This notion emphasizes the intersectional nature of violence and recognizes that individuals may experience multiple, interacting, and compounding forms of violence [32]. Particularly, displaced women are likely face polyvictimization as the trauma of war and displacement intersects with personal histories of abuse, including IPV and other forms of gender-based violence [32, 33]. Such prolonged exposure to multiple forms of violence can have a significant impact on mental health, increasing the risk of depression, anxiety, PTSD, and other mental health disorders [34, 35].

Considering these factors, the present research focuses on exploring polyvictimization among Iraqi and Syrian refugee women. More specifically, this study attempts to examine the relationship between different forms of childhood maltreatment and IPV victimization, taking into account other traumatic events such as war, displacement, and events in their daily lives. Drawing parallels with global data [36], we hypothesize that experiences of childhood maltreatment are significantly associated with IPV exposure, indicating increased risk of re-victimization and ongoing cycles of violence.

## Methods

### Participants

The study sample consisted of 332 displaced married women living in the Arbat displacement camps in Iraqi Kurdistan. The age of the participants at the time of the interview ranged from 20 to 62 years (*M* = 38.68, *SD* = 7.05). Participants’ ages at the time they married ranged from 11 to 33 (*M* = 18.77, *SD* = 3.83), and the number of children per participant ranged between one and fourteen (*M* = 5.73, *SD* = 2.26). A significant proportion of participants (81.3%) were in marriages arranged by their parents. Approximately three in five women (59.6%) were married to relatives. In terms of employment status, the vast majority of women (96.7%) reported no employment and identified themselves primarily as homemakers. Additional details on the socio-demographic characteristics of the participants are provided in Table 1.

### Procedure

Data for this study were collected through a comprehensive quantitative triadic family survey of displaced families from Iraq and Syria living in the Arbat camps in the Sulaymaniyah governorate of the Kurdistan Region of Iraq (KRI). The survey was conducted in March and April 2019, with trained paraprofessionals conducting interviews in participants’ tents or homes. All interviewers had at least a bachelor’s degree in psychology, they were fluent in both Kurdish and Arabic, and had an in-depth understanding of the culture and experiences of Iraqi and Syrian women.

Participants were recruited through the aforementioned survey. Detailed participant recruitment procedures have been described elsewhere [37, 38]. To protect the safety and privacy of participants who may have experienced IPV, the interviews were not framed as domestic violence investigations, but rather as general surveys exploring trauma, mental health, and the living conditions of displaced families. Women selected to participate were explicitly informed individually prior to beginning of the interviews that they would be asked about their childhood experiences and current IPV issues, in addition to questions about their war and displacement experiences, and all women consented. To ensure their confidentiality, the interviews were conducted in private locations where no one else was present during the interviews.

A comprehensive risk management process was implemented to protect both participants and staff. If a woman disclosed severe violence or mental health issues during the interview, immediate counselling was provided by staff trained in case management and mental health first aid. Participants were also referred to additional health care providers as needed to ensure continuity of care. All participating women were given contact information for emergency services and violence prevention hotlines at the end of the interview. They were also given information on how to reach local support organizations both inside and outside the camps. This approach ensured that participants had access to resources to seek help and support beyond the scope of the research study. Approval for the research was obtained from the respective ethics committees of the University of Bielefeld in Germany (reference number EUB 2015-046) and Koya University in KRI (reference number SHETC-1). In addition, the study and its protocols were approved by the local government authorities, including the camp administration, the Directorate of Social Affairs (DoSA) of the Ministry of Labor and Social Affairs, and the Protection Office of the Joint Crisis Coordination Center (JCC) of the Ministry of Interior in the KRI. A detailed description of the research procedures, ethical considerations, and risk management is described elsewhere [37, 39].

### Instruments

The War and Adversity Exposure Checklist (WAEC) was used to assess potentially traumatic events that women had experienced in their lifetime [40]. The WAEC was specifically designed to capture the experiences of Middle Eastern populations, particularly those who have been displaced in Iraq and Syria. It includes 26 potentially traumatising events, including both war-related events, which comprise 10 items (such as witnessing armed combat or experiencing forced separation from family members due to war), and adverse life experiences, which comprise 16 items (including natural disasters and life-threatening illnesses). Participants were asked to indicate whether or not they had experienced each of these events. The Cronbach’s alpha coefficient for the WAEC in the current study was α = 0.77.

The Gendered Violence in Partnerships Scale (GVPS) was used to assess IPV reported by women within the past year [41]. The GVPS is a validated and reliable instrument developed specifically to measure IPV among Iraqi and Syrian women. It consists of 18 items that assess various aspects of IPV, including husbands’ controlling behaviors (e.g., controlling their clothing, preventing them from working or studying, and isolating them from family or friends), impulsive aggression, whether verbal or physical (e.g., name-calling, hitting, kicking, slapping, and punching), aggregated physical assaults (e.g., attempted strangulation, attempted murder, or assault with a weapon), and existential threats (e.g., threats of divorce or taking another wife/partner). Each item of the scale is answered with a binary yes or no response. The Cronbach’s alpha coefficient for the IPV checklist in this sample was α = 0.88.

Childhood maltreatment was assessed using a brief version of the Adverse Experiences Checklist at Home, which focuses on various negative events experienced at home [42]. The checklist consisted of 12 items related to physical, sexual, and emotional abuse and neglect by family members. Participants were asked to report whether they had ever experienced such events during their childhood. In addition, the checklist included two questions about the consequences of such maltreatment. Participants were asked whether they had ever been injured as a result of family violence and whether they had ever required medical treatment for injuries resulting from family violence. The Cronbach’s alpha coefficient in this sample was α = 0.86.

PTSD symptoms were assessed using the Kurdish and Arabic adaptations of the Posttraumatic Stress Disorder Checklist for DSM-5 (PCL-5) [43–45]. The PCL-5 consists of 20 items that are consistent with the DSM-5 diagnostic standards for PTSD. Each item on the PCL-5 is rated on a five-point scale ranging from 0 (indicating “not at all”) to 4 (indicating “extremely”). According to the Kurdish and Arabic versions of the PCL-5, the optimal cut-off score for a probable diagnosis of PTSD is 23 [43]. The reliability of the PCL-5, as measured by Cronbach’s alpha coefficient in the context of this study, was reported to be α = 0.94.

### Data analysis

The levels of different traumatic events experienced (war-displacement-related events, adverse life events, IPV, and childhood maltreatment) were assessed using descriptive statistics that analysed the frequency of each reported event. The prevalence of probable PTSD diagnosis was determined by applying a cut-off score of 23 on the Kurdish and Arabic version of PCL-5 and examining its frequency. Relationships between variables were examined using Spearman correlation coefficients.

Hierarchical regression analysis was conducted to investigate potential variables contributing to IPV experienced by women from their current husbands within the past year. In the first stage of the analysis, sociodemographic variables such as age, number of children, and the presence of kinship marriage were included. In the second stage of the regression model, we included the sum score of PTSD symptoms and the number of non-war adverse life events that women had previously experienced. In addition, sum scores of different forms of childhood maltreatment were also included in our model. Due to violations of the normality assumption for certain variables, such as the number of wives a husband has and the number of negative life events, bootstrapping was used in the regression analysis. This involved generating 1000 random resamples with replacement. Bootstrapping is a modern statistical approach specifically designed to correct for potential biases associated with non-normally distributed predictor and dependent variable data [46, 47] and to provide greater precision than traditional methods such as data transformations or classic nonparametric methods [48]. All analyses were performed using Statistical Package for the Social Sciences (SPSS) version 29 (IBM Corp.).

## Results

### IPV

Participating women reported that their husbands had perpetrated between 0 and 16 incidents of IPV in the past 12 months (*M* = 1.24, *SD* = 2.61). A total of 121 (36.4%) had experienced at least one act of IPV perpetrated by their husbands in the past 12 months.

**Fig. 1:**
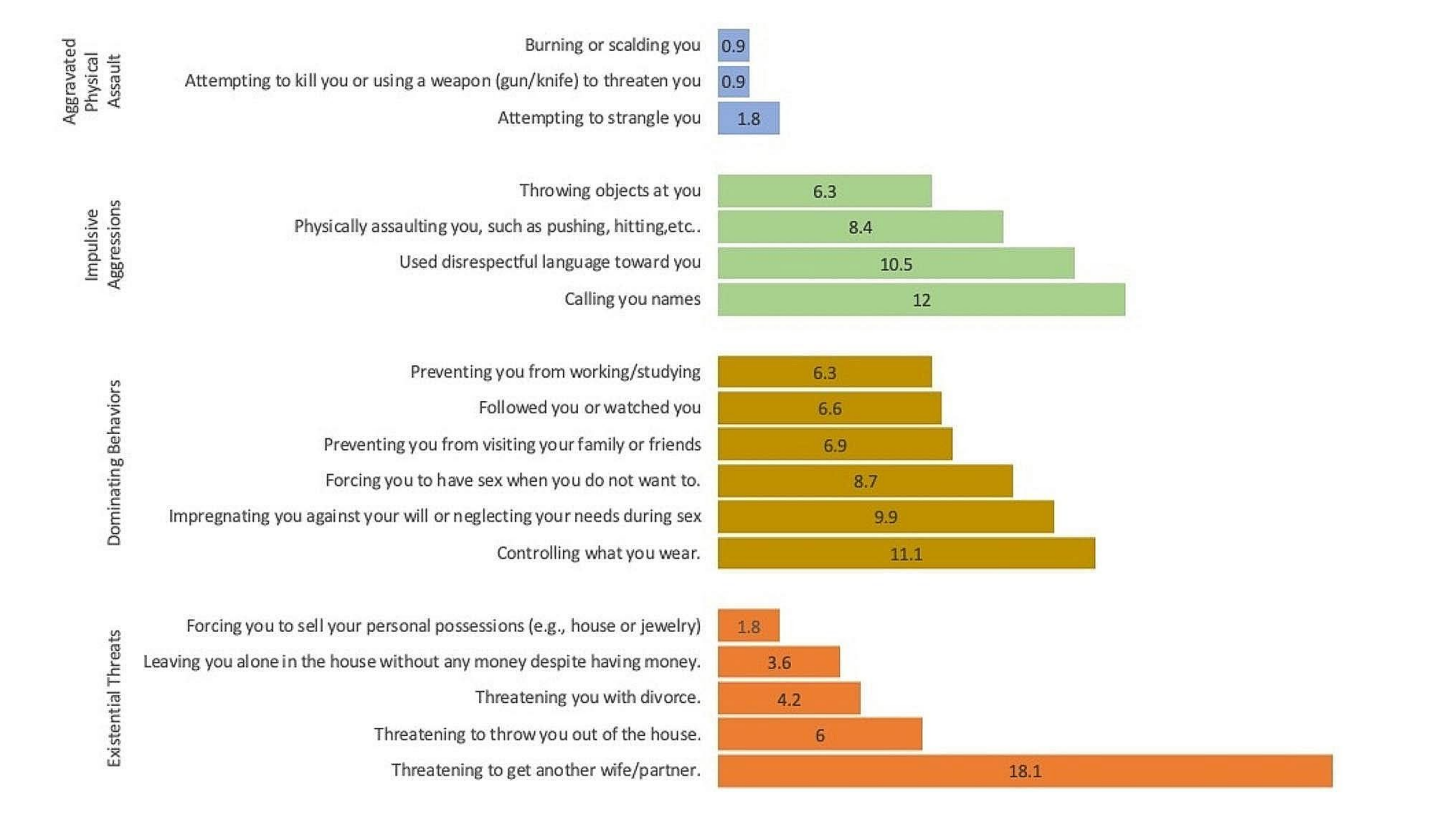
Percentage of IPV perpetrated by husbands in the past 12 months

Threats to remarry were the most commonly reported IPV event, affecting 18.1% of women. This was followed by name-calling, with an occurrence of 12%. Sexual objectification, which includes forcing a woman to become pregnant against her will or neglecting her needs during sex, was reported by 9.9% of women. Finally, physical violence, such as hitting, kicking, biting, punching, twisting arms, or pulling hair, was reported by 8.4% of participants (see Fig. 1).

### Childhood maltreatment

Participating women reported experiencing between 0 and 12 childhood maltreatment events (*M* = 1.11, *SD* = 2.08) perpetrated by their family members during their childhood. The most common forms of emotional maltreatment were verbal insults or yelling (24.4%) and verbal threats (19.6%). Physical abuse took several forms, including being hit with a hand (20.5%) or an object (8.1%) and having objects thrown at them (9.6%). An additional 2.7% reported experiencing sexual abuse, specifically rape, by immediate or extended family members during childhood. In addition, a small proportion of participants (0.6%) reported experiencing inappropriate touching of genitals by family members who were significantly older.

**Fig. 2:**
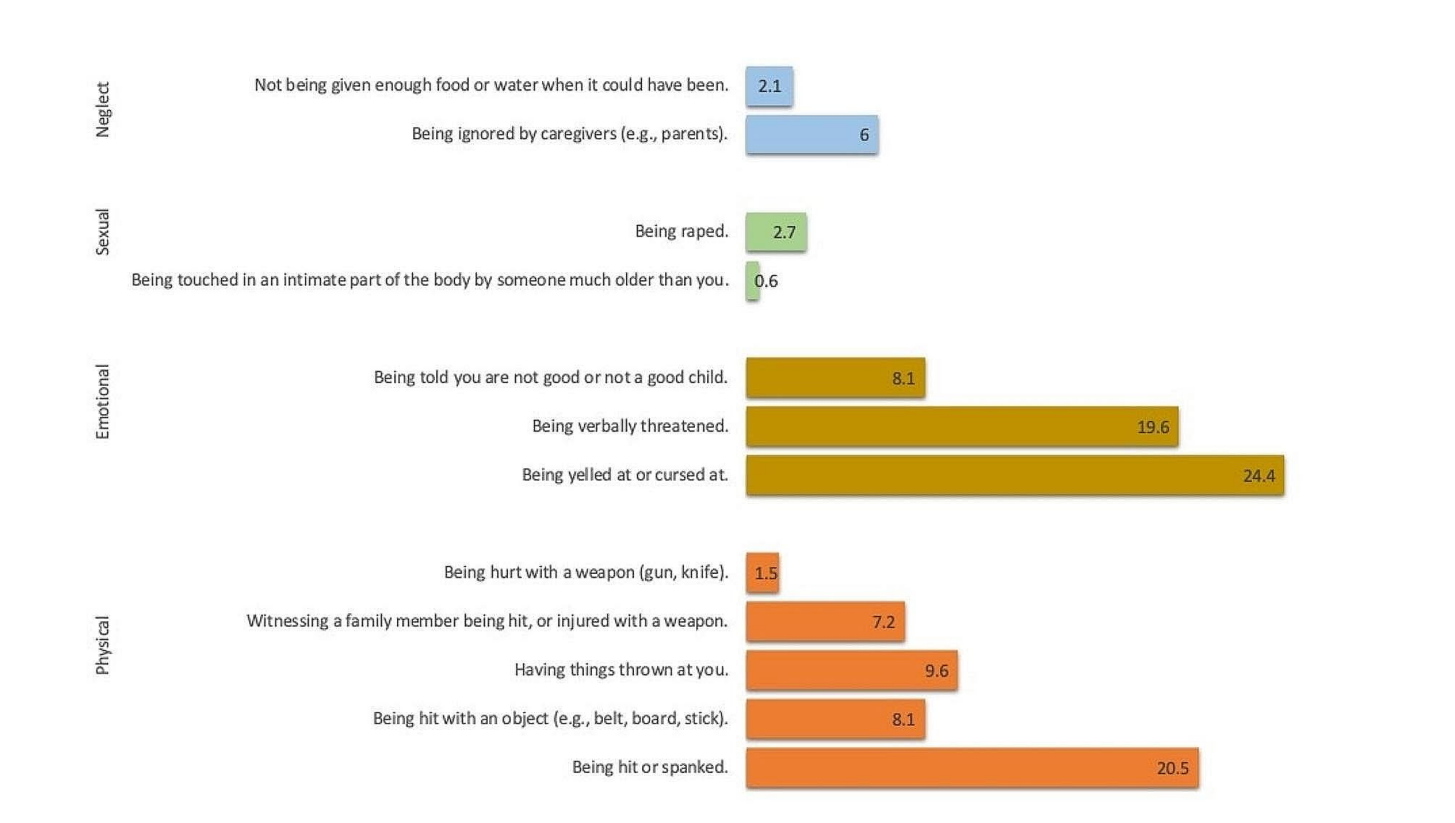
Percentage of reported childhood maltreatment perpetrated by family member

Childhood maltreatment resulted in serious physical injuries in 12% of participants, ranging from cuts and bruises to burns, fractures, and eye hematomas. In addition, 10.2% of participants reported the need for medical treatment due to the severity of the abuse they experienced. A detailed representation of the types of childhood maltreatment reported by participants is shown in Fig. 2.

### Traumatic events and PTSD symptoms

90.9% of participants had experienced at least one lifetime traumatic event (*M* = 4.37, *SD* = 3.29; range: 0–20). War-related events were the most commonly reported events, with 88.5% of participants having experienced at least one war-related traumatic event (*M* = 2.94, *SD* = 2.02; range: 0–10) and 58.1% having experienced at least one non-war and adverse lifetime traumatic event (*M* = 1.41, *SD* = 1.83; range: 0–16). The mean PTSD symptom score on the PCL-5 was 23.85 (*SD* = 17.79). Using the contextually validated cut-off score of the PCL-5, half (50.09%) of the participants met criteria for a probable PTSD diagnosis.

### Childhood maltreatment, trauma and PTSD symptoms predict IPV

Results from correlation coefficients indicate that childhood maltreatment was significantly correlated with IPV, number of non-war adverse life events, and symptoms of PTSD (*r*_*s*_ = 0.39, *r*_*s*_ = 33, and *r*_*s*_ = 0.33, respectively; *p*s < 0.001), presented in Table 2. To predict last year’s IPV, using hierarchical regression analysis, potential predictors of IPV, including sociodemographic variables, lifetime history of trauma, symptoms of PTSD, and childhood maltreatment were examined. A significant association was found between increased IPV and polygamy. This was demonstrated by the number of additional wives a husband had. Conversely, variables such as kinship-based marriage and number of children were negatively correlated with levels of IPV. Furthermore, among the various types of childhood maltreatment, only emotional maltreatment was identified as a robust indicator of IPV (Table 3. presents a bootstrapped multiple hierarchical regression analysis for the prediction of IPV among the study sample).

## Discussion

This study explores the relationship between exposure to childhood maltreatment and IPV victimization in adulthood among refugee women. The findings reveal that among displaced women interviewed in a refugee camp in northern Iraq, exposure to childhood maltreatment was associated with IPV victimization experienced in the past year. However, when accounting for trauma, PTSD, and demographic variables, only emotional childhood maltreatment emerged as a statistically significant predictor of IPV.

With 36% of participants reporting IPV in the past year, the results of our study are nearly four times higher than what a recent global review based on WHO data estimated - about 10% of women aged 15 and older experienced violence from an intimate male partner in the past year [49]. While there is a lack of meta-analyses providing accurate rates of IPV specifically against women in the Arab region, systematic reviews reveal that IPV rates vary significantly across Arab nations [50, 51] indicating a range of different social and socioeconomic factors influencing IPV occurrence in these countries. Examining the long-standing histories of conflict, political instability, and economic hardship in the region is necessary to understand IPV against women in Iraq and Syria. Notable events, including the Iran-Iraq War, Gulf Wars, US sanctions in 1990, the 2003 US invasion, post-Arab Spring war and displacement, and the rise of the Islamic State terrorist group, have significantly impacted the socioeconomic landscapes of the two countries. It is likely that these events have contributed to the emergence of diverse violent patterns including violence against women.

Research from other conflict-affected regions indicates a connection between war-induced forced displacement and a higher likelihood of IPV [52–54]. The instability of socio-economic conditions during war can shift power dynamics within households. For example, men may lose their primary role as breadwinners or suffer psychological trauma as a result of the conflict, which may lead to higher rates of IPV against women [30, 55–57]. Furthermore, living in refugee camps can increase women’s vulnerability to IPV. The refugee camp environments often present barriers to victims seeking formal protection and support, as broader family and community structures are disrupted. Furthermore, amidst the complexity of supply issues around basic needs, there seems to be a significant gap in the camps residents’ awareness of available protection services in the Iraqi and Syrian refugee context. While an increasing number of national and international NGOs are working with the government of Iraqi Kurdistan to address IPV, women often find it difficult to identify the right resources, leading to uncertainty about which organisations to approach for support. There seems to be an urgent need to establish clear and accessible service-mapping systems to ensure that all individuals, regardless of their educational background, can effectively identify and connect with service providers. In addition, while the study didn’t look in depth at the reasons why women might not report IPV to the authorities, feedback from the local research assistants who conducted the study’s interviews suggests that social stigma, fear and a lack of knowledge about the support available might prevent them from doing so.

The correlation and regression analyses showed significant statistic associations between exposure to childhood maltreatment, symptoms of PTSD, and IPV. This finding is consistent with numerical meta-analyses and systematic reviews, which indicated that exposure to childhood maltreatment increases the likelihood of mental health problems. For example, Norman et al. (2012) systematically reviewed and analysed 124 studies on the long-term mental health effects of child maltreatment and found indications for a causal relationship between child maltreatment and various mental health disorders, substance abuse, and suicide attempts [58]. These findings were also supported by a meta-analysis conducted by Gardner et al. (2019) on the association between child maltreatment and PTSD symptoms, which found that individuals exposed to child maltreatment had a threefold increased risk of PTSD [59]. Additionally, our study reveals that among various forms of childhood maltreatment, emotional childhood maltreatment is strongly associated with current PTSD symptoms. This finding underscores the significant psychological impact of childhood emotional maltreatment. Previous research has also confirmed this association, showing that emotional maltreatment has a significant and unique impact on adult mental well-being, over and above the effects of other forms of maltreatment. For instance, a recent meta-analysis and systematic review that included studies from the general population across various geographic regions found that emotional childhood maltreatment was associated with an increased risk of adulthood mental health problems, including depression, anxiety, PTSD, and other psychological symptoms [60]. Likewise, the correlation between childhood maltreatment and IPV aligns with earlier findings from meta-analyses (for review see [61–63]) indicating that experiencing maltreatment during childhood, and emotional maltreatment in particular, increases the likelihood of victimization by an intimate partner.

Although an association between past childhood maltreatment and recent IPV was observed in this study, it does not establish a causal relationship or clarify the potential mechanisms underlying the lasting psychological effects of childhood maltreatment, such as PTSD and revictimization. Meta-analyses suggest that child maltreatment may lead to emotional dysregulation, thereby enhancing the probability of developing PTSD and IPV in adulthood, particularly after experiencing other traumatic events [64, 65]. Longitudinal research indicates that emotion dysregulation is a significant predictor of the severity of common PTSD symptoms, such as intrusive memories, flashbacks, and distressing nightmares [66, 67]. Furthermore, emotional maltreatment disrupts attachment formation, leading to difficulty in developing secure relationships and obtaining support during times of distress [68]. This may also explain this study’s findings.

Our study has identified specific family characteristics and structures that are associated with IPV. Significantly, our findings demonstrate that women who marry at a young age and those who are in polygamous relationships are more likely to experience IPV. Although the practice of early marriages and polygamy is often allowed by legal and cultural norms in both Iraqi and Syrian societies, it is important to note that young brides may lack the resources and independence to address or escape from abusive partners. This observation is particularly relevant in less privileged regions of Iraq and Syria, where social norms and economic constraints are often incentives for early marriage [69]. In addition, the nature of polygamous relationships can lead to competition, jealousy, and complex power dynamics, all of which can potentially increase IPV [70].

Interestingly, our study found a significant link between having more children and experiencing less IPV among displaced Arab and Kurdish women. One explanation for this could be the cultural importance placed on family ties in Iraq and Syria, where larger families may strengthen these ties [71]. Such families often have a stronger sense of unity, resulting in shared household and childcare responsibilities among siblings. Notably, older children in Iraqi and Syrian households often contribute financially, which may reduce the likelihood of triggering IPV between the parents. In Iraqi and Syrian cultural traditions, marriage between close relatives, particularly cousins, is common. The results of the current study suggest that these marriages are associated with lower rates of IPV, possibly due to strong family support systems and established family ties. Further research is needed to clarify the influence of this cultural practice on family structure, family cohesion, and the quality of partner relationships.

This study addresses a commonly overlooked issue in war-traumatized and displaced populations by examining associations between childhood maltreatment and current IPV among displaced women from Syria and Iraq. The findings provide important insights into the challenges faced by women in refugee camps. The strength of the study is enhanced by the involvement of trained female psychologists in conducting interviews and the application of valid and reliable screening instruments. This methodology created a supportive environment for discussing sensitive topics such as domestic violence, which likely enhanced the depth and comprehensiveness of the data. However, a number of limitations of this study have to be noted. Even with trained local interviewers’ involvement, participants’ responses might be influenced by deep-seated feelings of shame, mistrust, and the social stigma related to domestic violence. These deep-rooted emotions may impact the accuracy of reported childhood maltreatment and IPV rates. Moreover, the cross-sectional design of our study limits our ability to determine causal relationships among childhood maltreatment, IPV, and mental health issues. The assessment instruments used in this study to evaluate IPV and childhood maltreatment had limitations that may have limited the depth and precision of the conclusions drawn from our findings. These instruments did not provide a complete evaluation of the frequency and severity of different types of abuse. Rather, they focused on identifying whether women had experienced abuse or not. Understanding the frequency, chronicity, and severity of abusive acts is crucial for understanding the intricacies of marital dynamics and for distinguishing between different forms of abuse, such as intimate terrorism and isolated incidents in the context of conflict. Also, the specific displacement context in Iraq limits the generalizability of our findings to other populations. The study also did not explore potential mediating or moderating factors between childhood maltreatment and its long-term negative impacts, such as emotional regulation or attachment styles or perceptions of violence which should be researched using qualitative methods as well.

Future research initiatives should use a longitudinal methodology that integrates both qualitative and quantitative approaches to better understand the long-term effects of childhood trauma on mental health and the risk of re-victimization in adulthood. In addition, our understanding of these lasting effects will be enhanced by studying diverse cultural populations.

Moreover, in a practical term, addressing the needs of refugee populations suffering from IPV requires a multidimensional and socio-ecological approach that considers individual, family and structural level factors. At the individual level, all psychosocial interventions should be culturally sensitive and trauma-informed, recognizing that refugees’ experiences may be influenced by their cultural backgrounds, historical trauma, and their own individual past trauma. In addition, it is essential to provide access to mental health support and counselling services that are tailored to the specific needs of each woman survivor of IPV and child maltreatment. Interventions at the family level should focus on addressing the dynamics within refugee families affected by IPV. This can be facilitated through family counselling, parenting support, and educational programs aimed at promoting healthy relationships and communication. Furthermore, survivors should be empowered within their families and communities and provided with the necessary tools to break the cycle of violence. Lastly, interventions should address the systemic challenges faced by refugee women at the structural and societal levels. These challenges include limited access to health care, legal barriers, and social isolation. Collaboration with community organizations, advocacy groups, and policymakers is essential to creating an empowering environment that facilitates women refugees’ access to resources, support, and protection.

## Conclusion

The current study highlights the multifaceted interplay between childhood maltreatment and IPV within the specific sociocultural context of displaced women from Iraq and Syria. While the findings are consistent with previous literature on impacts of childhood trauma, they also highlight the need for in-depth scientific research and the formulation of interventions to address the unique needs and challenges of displaced women and their communities.

**Table 1:** Sociodemographic characteristics

Age in years, mean (SD)^a^		38.68 (7.05)
Formal education in years, mean (SD) ^b^		3.62 (3.61)
Individual monthly income in IQD, mean (SD) ^c^		20422.96 (95485.05)
Number of children, mean (SD)^d^		5.73 (2.26)
Marriage age in years, mean (SD)^e^		18.77 (3.83)
The number of other wives that the husband has beside the one interviewed, mean (SD) ^f^		0.21 (0.53)
Polygamy	Yes, n (%)	50 (15.1)
	No, n (%)	282 (84.9)
Ethnicity	Kurd, n (%)	179 (53.9)
	Arab, n (%)	153 (46.1)
Nationality	Iraqi, n (%)	172 (51.8)
	Syrian, n (%)	160 (48.2)
Ways to get married	Parents’ choice, n (%)	270 (81.3)
	Exchange, n (%)	57(17.2)
	Forced, n (%)	4 (1.2)
	Running away, n (%)	1 (0.3)
Kinship Marriage	Yes, n (%)	198 (59.6)
	No, n (%)	134(40.4)
Occupation	Housewife, n (%)	321 (96.7)
	Full-time work, n (%)	7 (2.1)
	Part-time work, n (%)	4 (1.2)

Note: ^a^ age range 20–62. ^b^ score range 0–20. ^c^ score range 0–1,000,000. ^d^ score range 1–14. ^e^ score range 11–33. ^f^ score range 0–3

**Table 2:** Interrelationships between experiences of childhood maltreatment, PTSD symptoms, and other forms of violence

		1	2	3	4	5	6	7	8
1. Any type of childhood maltreatment			0.94**	0.87**	0.28**	0.48**	0.39**	0.33**	0.33**
2. Emotional				0.76**	0.20**	0.44**	0.36**	0.28**	0.329**
3. Physical					0.25**	0.41**	0.33**	0.33**	0.29**
4. Sexual						0.26**	0.19**	0.12**	0.080
5. Neglect							0.18**	0.21**	0.23**
6. IPV								0.28**	0.29**
7. Number of negative life events (non-war)									0.33**
8. PTSD									

** *P* < .001, IPV = intimate partner violence

**Table 3:** Bootstrapped multiple hierarchical regression analysis of IPV among displaced women

Model	Predictor	b (SE)	95% CI (LB, UB)	*P* value
Step 1				
	Age	0.031 (0.03)	(-0.029, 0.091)	> 0.05
	Number of children	− 0.216 (0.081)	(-0.364, − 0.056)	< 0.01
	Kinship marriage	− 0.667 (0.289)	(-1.287, − 0.137)	< 0.05
	The number of wives that husband has	0.679 (0.221)	(0.260, 1.127)	< 0.01
Step 2				
	PTSD symptoms	0.024 (0.010)	(0.005, 0.042)	< 0.05
	Number of negative life events (non-war)	0.191 (0.109)	(-0.027, 0.389)	> 0.05
	Number of child maltreatment events.			
	Emotional	1.024 (0.295)	(0.443, 1.593)	< 0.001
	Physical	0.182 (0.338)	(-0.447, 0.882)	> 0.05
	Sexual	1.058 (2.096)	(-2.068, 6.773)	> 0.05
	Neglect	− 0.666 (0.917)	(-2.180, 1.439)	> 0.05

Variance explained by full model: R^2^ = 0.35; *F* 10, 321 = 17.25; *P* < .001

## Data Availability

The datasets generated and/or analyzed during the current study are not publicly available due to the terms of consent agreed upon by the participants. However, they are available from the corresponding author upon reasonable request.

## Abbreviations

IPV: Intimate Partner Violence
PTSD: Post-Traumatic Stress Disorder
WAEC: War and Adversity Exposure Checklist
PCL-5: PTSD Checklist for DSM-5
GVPS: Gendered Violence in Partnerships Scale
KRI: Kurdistan Region of Iraq
